# Uncovering the Mechanisms and Molecular Targets of Weibing Formula 1 against Gastritis: Coupling Network Pharmacology with GEO Database

**DOI:** 10.1155/2021/5533946

**Published:** 2021-08-21

**Authors:** Ke Chen, Luojian Zhang, Zhen Qu, Feng Wan, Jia Li, Ye Yang, Hui Yan, Shile Huang

**Affiliations:** ^1^Department of Traditional Chinese Medicine, People's Hospital of Xinjin District, Chengdu, China; ^2^Department of Rehabilitation Medicine, China MCC5 Group Corp. Ltd. Hospital, China; ^3^Department of Foot and Ankle, Sichuan Provincial Orthopaedic Hospital, China; ^4^School of Basic Medical Sciences, Chengdu University of Traditional Chinese Medicine, China; ^5^State Key Laboratory of Southwestern Chinese Medicine Resources/Chengdu University of Traditional Chinese Medicine, China; ^6^Department of Acupuncture and Moxibustion, Hospital of Chengdu University of Traditional Chinese Medicine, China

## Abstract

Weibing Formula 1, a classic traditional formula, has been widely used clinically to treat gastritis in recent years. However, the potential pharmacological mechanism of Weibing Formula 1 is still unclear to date. A network pharmacology-based strategy was performed to uncover the underlying mechanisms of Weibing Formula 1 against gastritis. Furthermore, we structured the drug-active ingredients-genes–disease network and PPI network of shared targets, and function enrichment analysis of these targets was carried out. Ultimately, Gene Expression Omnibus (GEO) datasets and real-time quantitative PCR were used to verify the related genes. We found 251 potential targets corresponding to 135 bioactive components of Weibing Formula 1. Then, 327 gastritis-related targets were known gastritis-related targets. Among which, 60 common targets were shared between potential targets of Weibing Formula 1 and known gastritis-related targets. The results of pathway enrichment analysis displayed that 60 common targets mostly participated in various pathways related to Toll-like receptor signaling pathway, MAPK signaling pathway, cytokine-cytokine receptor interaction pathway, chemokine signaling pathway, and apoptosis. Based on the GSE60427 dataset, 15 common genes were shared between differentially expressed genes and 60 candidate targets. The verification results of the GSE5081 dataset showed that except for DUOX2 and VCAM1, the other 13 genes were significantly upregulated in gastritis, which was consistent with the results in the GSE60427 dataset. More importantly, real-time quantitative PCR results showed that the expressions of PTGS2, MMP9, CXCL2, and CXCL8 were significantly upregulated and NOS2, EGFR, and IL-10 were downregulated in gastritis patients, while the expressions of PTGS2, MMP9, CXCL2, and CXCL8 were significantly downregulated and NOS2, EGFR, and IL-10 were upregulated after the treatment of Weibing Formula 1. PTGS2, NOS2, EGFR, MMP9, CXCL2, CXCL8, and IL-10 may be the important direct targets of Weibing Formula 1 in gastritis treatment. Our study revealed the mechanism of Weibing Formula 1 in gastritis from an overall and systematic perspective, providing a theoretical basis for further knowing and application of this formula in the future.

## 1. Introduction

Gastritis is digestive diseases with high incidence, which is mainly manifested as variety of pathogenic factors invading the gastric mucosal epithelium, resulting in persistent chronic inflammatory changes [1]. Gastritis is mainly caused by Helicobacter pylori infection, drug induction by nonsteroidal anti-inflammatory drugs, smoking, alcohol consumption, and other factors [2, 3]. Gastritis is classified into acute gastritis and chronic gastritis according to the duration of disease [4]. Although the incidence of chronic gastritis has declined in recent decades, it remains a serious medical problem worldwide [5]. Gastritis has been recognized as a precancerous lesion of gastric cancer [6]. Despite current treatments such as antacids, H-2 blockers, proton pump inhibitors, and antibiotics can relieve some of the main symptoms of gastritis, and these drugs can also cause a series of serious side effects, including osteoporosis, hypercarotenemia, constipation, and diarrhea [2]. Therefore, it is urgently to exploit novel and safe therapeutic strategies based on traditional medicinal plants.

Traditional Chinese medicine is a complementary therapy that plays a crucial role in healthcare of Asian population and is gradually recognized in western countries due to the feasible therapeutic efficacy and minimal side effects [7, 8]. In traditional Chinese medicine theory, gastritis is divided into dampness obstructing the spleen–stomach, spleen–stomach Qi deficiency, spleen–stomach deficiency cold, liver–Qi stagnation, and stagnant heat in the liver–stomach based on the accumulation pattern of damp heat in spleen and stomach [9]. Weibing Formula 1 is composed of twelve Chinese medicines, including Herba Taraxaci (Chinese name: Pugongying), Plantaginis Herba (Chinese name: Cheqiancao), Bletilla striata (Chinese name: baiji), Crataegus pinnatifida Bunge (Chinese name: Shanzha), Areca Catechu L (Chinese name: Dafupi), Aucklandiae Radix (Chinese name: Muxiang), Paeoniae Radix Alba (Chinese name: Baishao), Aurantii Fructus (Chinese name: Zhike), Atractylodes Macrocephala Koidz (Chinese name: Baishu), Arecae Semen (Chinese name: Binlang), Coptidis Rhizoma (Chinese name: Huanglian), and Licorice (Chinese name: Gancao). In China, Weibing Formula 1 is often used to treat gastritis and has achieved good therapeutic effects. However, the pharmacological mechanism of Weibing Formula 1 in the treatment of gastritis remains understood.

**Figure 1:**
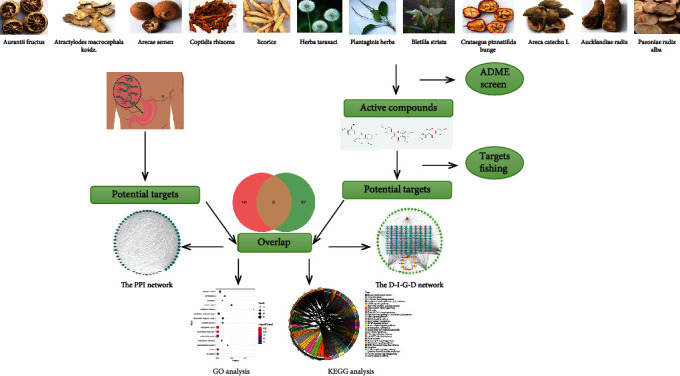
The flowchart of network pharmacology-based strategy for deciphering the mechanisms of Weibing Formula 1 acting on gastritis.

Network pharmacology is a novel approach to discover and develop traditional medicinal drug. Until now, this way has been successfully used to clarify the multitarget effects of traditional Chinese medicine in various diseases [10–13]. Network pharmacology has effectively bridged the gap between Chinese medicine and western medicine and greatly promoted the research on the synergistic mechanism of traditional Chinese medicine and western medicine [14]. Therefore, the goal of this study was to illustrate the intrinsic mechanisms of Weibing Formula 1 in the treatment of gastritis through network pharmacology analysis. We first screened one database for bioactive components of Weibing Formula 1 and targets prediction, followed by searching of known Weibing Formula 1-related targets, construction of drug-active ingredients-genes–disease, and PPI network; the function enrichment analysis of the overlap targets between Weibing Formula 1 and gastritis, in turn. In addition, we used the GEO data to verify differentially expressed genes in the overlap targets between Weibing Formula 1 and gastritis and obtained several key pathways through gene set enrichment analysis. Furthermore, the GSE5081 dataset was used to further validate the 15 common genes between the differentially expressed genes and 60 candidate targets. Finally, we carried out the real-time quantitative PCR analysis to verify several overlap targets between Weibing Formula 1 and gastritis. The flowchart of the experimental procedures in this study is illustrated in Figure 1.

## 2. Materials and Methods

### 2.1. Identification the Bioactive Ingredients of Weibing Formula 1

Weibing Formula 1 is prepared by combining twelve herbs, such as Plantaginis Herba, Bletilla striata, Crataegus pinnatifida Bunge, Areca Catechu L, Aucklandiae Radix, Paeoniae Radix Alba, Aurantii Fructus, Atractylodes Macrocephala Koidz, Arecae Semen, Coptidis Rhizoma, Licorice, and Herba Taraxaci. The bioactive compounds of twelve herbs were collected from the traditional Chinese medicine system pharmacology database (TCMSP, https://tcmspw.com/tcmsp.php) [15].. As a pharmacological database, TCMSP contains a large number of Chinese herbal medicines, active ingredients, and their targets, including pharmacokinetic parameters of some active compounds, such as oral bioavailability (OB), drug-likeness (DL), permeability of Caco-2, and blood brain barrier. OB and DL are important indicators to assess absorption, distribution, metabolism, and excretion characteristics of drugs and are also key factors to screen active components of drugs. To better uncover the active compounds of Weibing Formula 1 more comprehensively, the compounds in Weibing Formula 1 with OB ≥ 30% [16] and DL ≥ 0.18 [17] were selected as candidate ingredients for following analyses in the current study.

### 2.2. Target Search

The active ingredients of traditional Chinese medicine play a role in related biological functions through regulating targets. The protein targets of the active ingredients of 12 herbs of Weibing Formula 1 were obtained by the TCMSP database based on ligand prediction method. We then deleted redundant information and reserved only those targets that could interact directly with each of these ingredients in Weibing Formula 1. In addition, the protein names of the Weibing Formula 1 bioactive ingredients were restricted to gene using the UniProt (https://www.uniprot.org/) database [18], and species was inverted to“Homo sapiens” so that name standardization and deduplication could be achieved based on the UniProt number [19]. The compound targets having no satisfaction with the selection were deleted. We combined the data to obtain the gene symbol and finally acquired the possible target of each herb in Weibing Formula 1.

### 2.3. Identification of Gastritis-Related Targets

The gastritis-related targets were collected from DisGeNET (https://www.disgenet.org/home/), MalaCards (http://www.malacards.org/), and CTD (http://ctdbase.org/). DisGeNET is a well-established resource to address the genetic basis of human diseases [20]. MalaCards is a human disease database that combines 68 databases and contains information about 20,000 diseases [21]. In order to acquire the gastritis-related targets, we searched in the DisGeNET and MalaCards databases with the keyword “gastritis” to search the targets related to gastritis. The Comparative Toxicogenomics Database (CTD) is a powerful and open database that provides manual management information on chemical-gene/protein interactions, chemical-disease, and gene-disease relationships [22]. The platform of CTD was retrieved using the keyword “gastritis,” and the gastritis-related targets were identified based on a score ≥ 40 and reference count ≥ 15. We removed the overlapping targets in the three databases DisGeNET, MalaCards, and CTD and finally obtained the targets related to gastritis.

### 2.4. Construction of Drug-Active Ingredients-Genes–Disease Network

First, we used Venn diagrams to analyze the intersection of drug targets and disease-related genes. Then, we constructed a network of complex information based on interactions between the drug (Weibing Formula 1), active ingredients, gene, and disease (gastritis). Ultimately, the Cytoscape v3.7.1 (http://www.cytoscape.org/) was used to visualize the drug-active ingredients-genes–disease network.

### 2.5. Protein–Protein Interaction (PPI) Network Construction

Based the above analyses, the on the overlap targets of Weibing Formula 1 and gastritis were used to build the PPI network. The STRING database (https://STRING-db.org/) is a database for searching known protein interactions and predicting protein interactions, including direct interactions and indirect interactions. Then, the overlap targets were imported into the STRING database to acquire PPI with the species limited to “Homo sapiens.” Ultimately, the PPI network of these targets was visualized using the Cytoscape V3.7.1. The hub nodes were identified in each network by calculating the size of the degree.

### 2.6. Function Enrichment Analysis

Gene Ontology (GO) analysis and KEGG pathway enrichment analysis were carried out using the David 6.8 (https://david.ncifcrf.gov/). Functional categories with *p* value <0.05 were considered significant.

### 2.7. Real-Time Quantitative Polymerase Chain Reaction (PCR) Analysis

Twenty-three blood samples of 8 normal control, 7 gastritis patients, and 8 gastritis patients treated with Weibing Formula 1 were obtained. This study was approved by the Institutional Ethics Committees of our hospital. All the participants had signed a written informed. Total RNA was extracted from samples using a RNA simple total RNA kit (Thermo Scientific, Waltham, MA, USA). RNA was reverse-transcribed with HiScript™ Reverse Transcriptase (Vazyme, Beijing, China) following the manufacturer's instructions. Quantitative real-time PCR was conducted using the SYBR™ Green Master Mix (Vazyme) on QuantStudio 6 Flex system. The 2-∆∆Ct method was the basis for analyze the relative quantification of genes. The human ACTB was used as endogenous controls for gene expression in analysis.

### 2.8. GEO Data Preprocessing and Identification of Differentially Expressed Genes

We searched datasets from the Gene Expression Omnibus (GEO) database (http://www.ncbi.nlm.nih.gov/geo/) with the keywords “gastritis” [MeSH Terms] OR “gastritis” [All Fields]) AND “Homo sapiens”[porgn] AND “gse”[Filter]. The inclusion criteria for the present study were as follows: (1) the type of dataset was described as expression profiling by array. (2) Dataset should be whole-genome mRNA expression profile by array. (3) Datasets were obtained by tissue samples of gastritis and normal control group. (4) The datasets should be normalized or original, and xx sets of mRNA data were selected. The exclusion criteria for this study were as follows: (1) cell line and animal level studies and (2) sample size less than 30. Eventually, two datasets (GSE60427 and) were included in this study. The GSE60427 was downloaded from the GEO database (https://www.ncbi.nlm.nih.gov/geo/, Platform: GPL17077, 8 normal and 24 gastritis samples). First, the R software (v.3.6.3) was used for the identification of differentially expressed genes between the gastritis and normal samples. Then, the Benjamini-Hochberg method was used to improve the false discovery rate (FDR) in multiple comparisons. Furthermore, FDR value < 0.01 and ∣log fold change | >1 as the cut-off values were deemed statistically significant. The volcano map, Venn diagram, and heat map of the differentially expressed genes were produced using R package, Venny 2.1.0 and R package, respectively. GSE5081, previously researched using GPL570 platform of Affymetrix Human Genome U133 Plus 2.0 Array, included 16 control and 16 gastritis samples and was used to further validate the GSE60427 results.

### 2.9. Gene Set Enrichment Analysis

The samples in the GSE60427 dataset were divided into two groups including 24 gastritis patients and 8 normal controls. Gene set enrichment analysis (http://www.broadinstitute.org/gsea/index.jsp) was performed to understand the meaningful KEGG pathway in the two groups. The annotated gene sets of version 6.0 were downloaded from the Molecular Signatures Database (MSigDB; http://software.broadinstitute.org/gsea/msigdb/index.jsp). The inclusion criteria were normalized *p* < 0.05 and FDR < 25%.

## 3. Results

### 3.1. Bioactive Components in Weibing Formula 1

Although there are thousands of ingredients in traditional Chinese medicine prescriptions, only a few them meet the satisfactory pharmacokinetic and pharmacodynamic characteristics that finally determine the efficacy [19, 23]. Based on the criteria of OB ≥ 30% and DL ≥ 0.18, a total of 135 bioactive ingredients of Weibing Formula 1 were obtained in the TCMSP database. The top 10 active compounds in target number were presented in Table 1. After excluding duplicates, 251 corresponding potential targets of these 135 bioactive components were chosen for further analysis.

### 3.2. Known Gastritis-Related Targets

**Figure 2:**
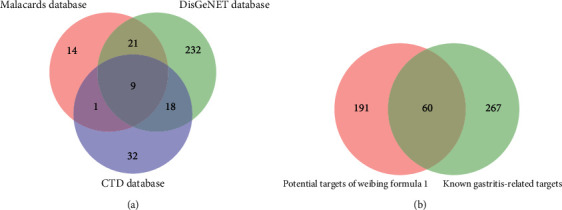
Venn diagram of overlapping gene symbols between the gastritis and Weibing Formula 1. (a) Venn diagram of known gastritis-related targets. (b) Venn diagram of shared targets between potential targets of Weibing Formula 1 and known gastritis-related targets.

Gastritis-related gene and protein targets were obtained from 3 databases, including 280 targets from DisGeNET, 45 targets from the MalaCards, and 60 targets from the CTD. After removing duplicates, 327 gastritis-related targets were retrieved (Figure 2(a)). Of which, 60 common targets were shared between potential targets of Weibing Formula 1 and known gastritis-related targets (Figure 2(b)). These 60 common candidate targets may be the key for gastritis treatment by Weibing Formula 1.

### 3.3. Drug-Active Ingredients-Genes–Disease Network Construction of Weibing Formula 1 in the Treatment of Gastritis

**Figure 3:**
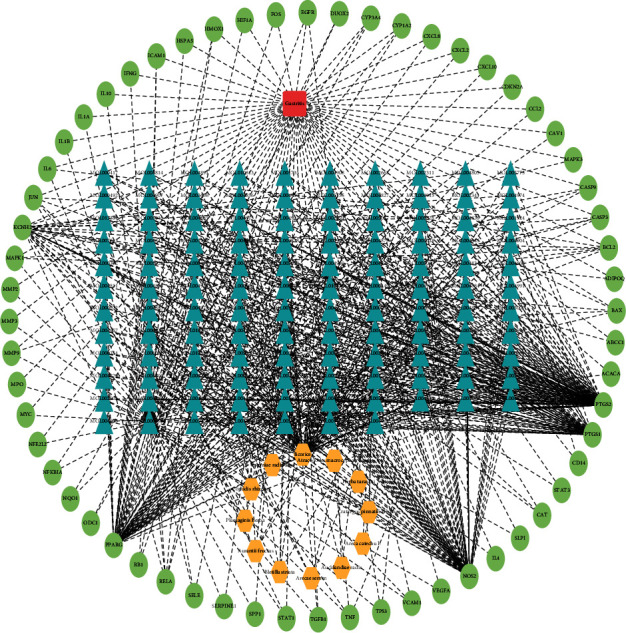
Drug-active ingredients-genes–disease network construction of Weibing Formula 1 in the treatment of gastritis. The saffron yellow node represents 12 traditional Chinese medicine ingredient of Weibing Formula 1, and the red node represents gastritis. The blue nodes represent the active ingredients in Weibing Formula 1. The green nodes represent the overlapping gene between the gastritis and Weibing Formula 1.

To understand how Weibing Formula 1 may act against gastritis, we utilized Cytoscape to structure a drug-active ingredients-genes–disease network (Figure 3). The saffron yellow node represented 12 traditional Chinese medicine ingredient of Weibing Formula 1, and the red node represents gastritis. Also, the 117 blue nodes represented the active ingredients in Weibing Formula 1. The 60 green nodes represented the overlapping gene between the gastritis and Weibing Formula 1. The edges indicated that nodes interacted with each other.

### 3.4. PPI Network Construction of 60 Candidate Targets

**Figure 4:**
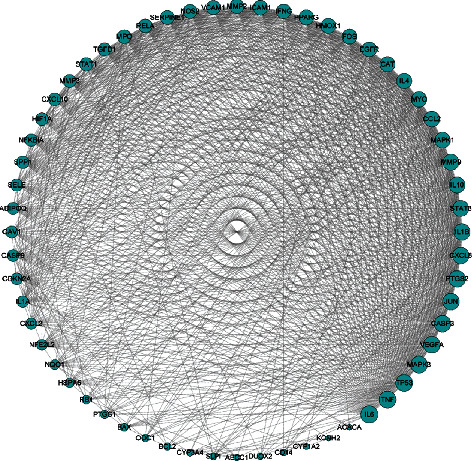
PPI network construction of 60 candidate targets. The nodes and edges represent interacting proteins and interactions, respectively.

PPI network is a relationship network between biomolecule and plays an important role in biological process [24]. Therefore, we constructed the PPI network to understand the therapeutic mechanism of Weibing Formula 1 in gastritis. In the STRING database, the PPI network of the 60 candidate targets was established. As shown in Figure 4, the PPI network consisted of 60 nodes and 951 edges. In the PPI network, nodes and edges represent interacting proteins and interactions, respectively.

### 3.5. Function Enrichment of 60 Candidate Targets

**Figure 5:**
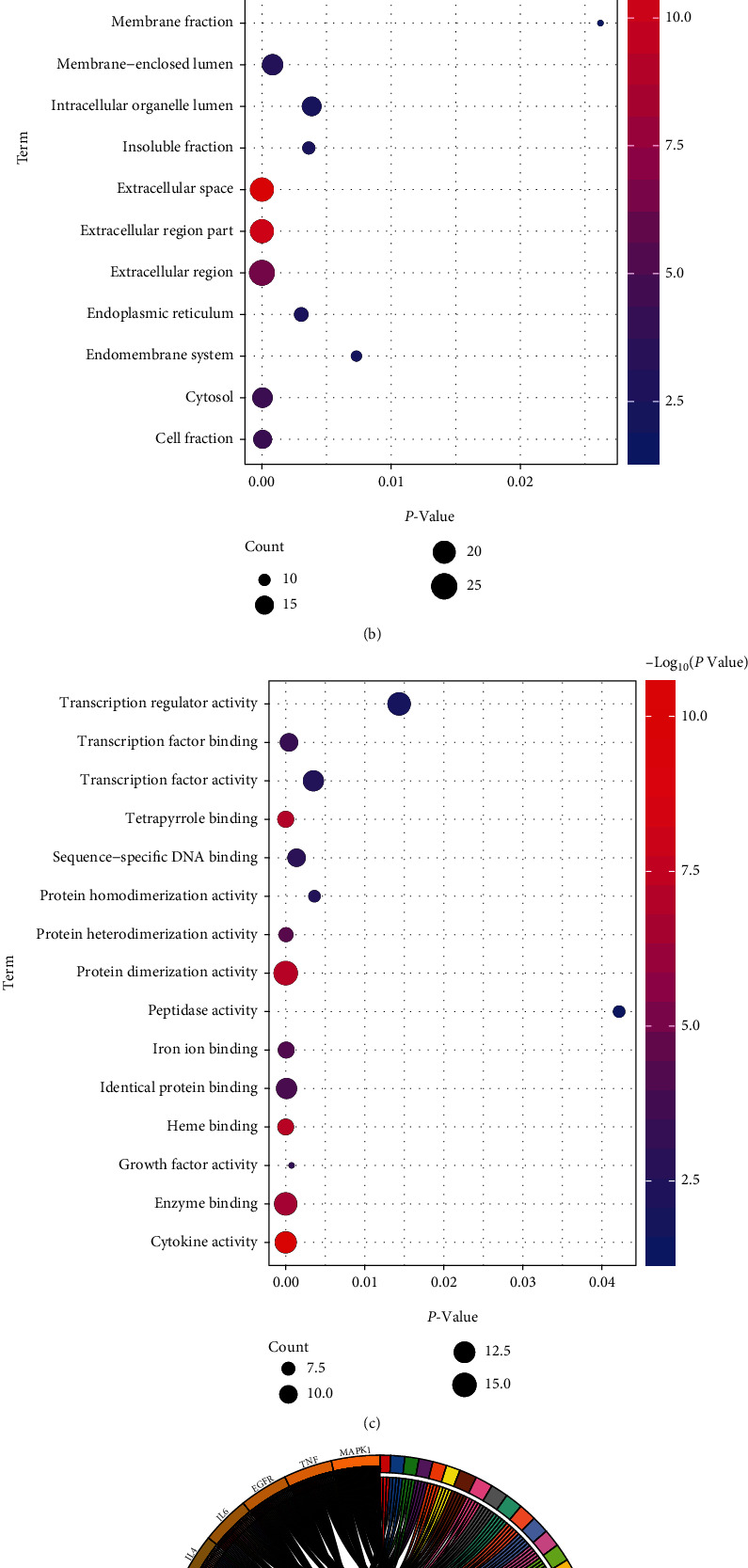
Functional enrichment analysis of 60 candidate targets. (a) Biological process. (b) Cellular component. (c) Molecular function. (d) KEGG pathways.

To further assess the 60 candidate targets, function enrichment analysis was carried out using the David 6.8. For GO enrichment analysis, these targets mainly enriched in the nuclear lumen, intracellular organelle lumen, nucleoplasm, and other regions of cell and were involved in regulation of transcription, DNA-dependment, regulation of RNA metabolic process, and other biological processes. They displayed multiple molecular functions, such as transcription regulator activity, transcription factor activity, and sequence-specific DNA binding. The top 15 of biological process, cellular component, and molecular function were demonstrated in Figures 5(a)–5(c), respectively. KEGG pathway enrichment analysis displayed that these candidate targets were markedly enriched in Toll-like receptor signaling pathway, NOD-like receptor signaling pathway, apoptosis, T cell receptor signaling pathway, MAPK signaling pathway, cytokine-cytokine receptor interaction pathway, and chemokine signaling pathway (Figure 5(d)).

### 3.6. GEO Data Verified Differentially Expressed Genes

**Figure 6:**
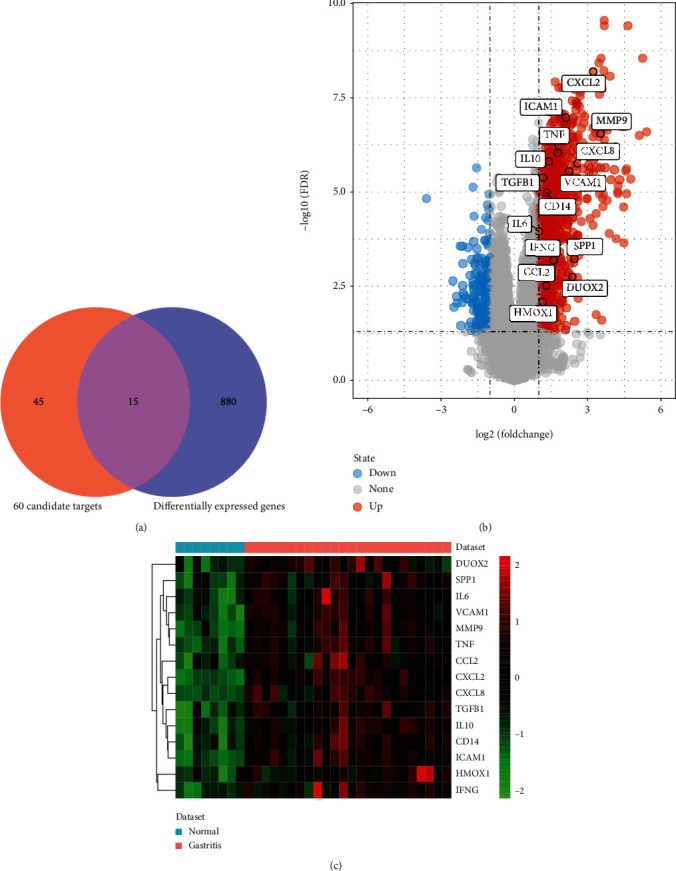
GSE60427 data verified differentially expressed genes. (a) Venn diagram of shared genes between differentially expressed genes and 60 candidate targets. (b) The volcano map of shared genes between differentially expressed genes and 60 candidate targets. (c) The heat map of shared genes between differentially expressed genes and 60 candidate targets.

**Figure 7:**
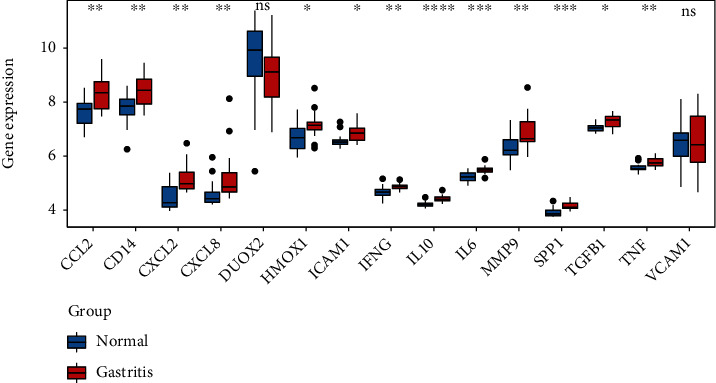
GSE5081 data verified the 15 common genes between the differentially expressed genes and 60 candidate targets. *X* axis represents different genes, and *Y* axis represents gene expression. ∗ indicated *p* value <0.05. ∗∗ indicated *p* value <0.01. ∗∗∗ indicated *p* value <0.001. ∗∗∗∗ indicated *p* value <0.0001.

GSE60427 was used to identify differentially expressed genes according to the criteria of FDR < 0.01 and ∣logfold change | >1. A total of 895 differentially expressed genes were obtained between gastritis and normal control, of which 783 genes were upregulated and 112 genes were downregulated. The top 20 differentially expressed genes were listed in Table 2. Among which, 15 common genes were shared between differentially expressed genes and 60 candidate targets (Figure 6(a)). The volcano map and heat map of 15 common genes were listed in Figures 6(b) and 6(c), respectively. In order to further verify the 15 common genes between the differentially expressed genes and 60 candidate targets, we verified them in the GSE5081 dataset. The results showed that except for DUOX2 and VCAM1, the other 13 genes were significantly upregulated in gastritis compared to normal (Figure 7), which was consistent with the results in the GSE60427 dataset.

**Figure 8:**
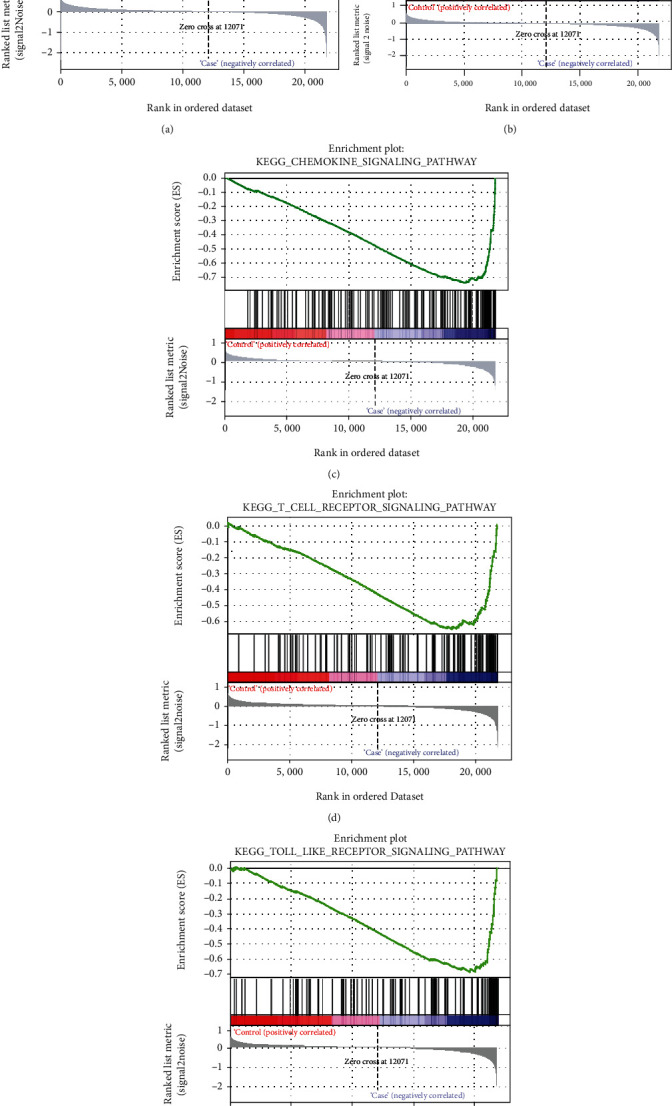
Enrichment plots from gene set enrichment analysis. (a) MAPK signaling pathway. (b) Cytokine-cytokine receptor interaction. (c) Chemokine signaling pathway. (d) T cell receptor signaling pathway. (e) Toll-like receptor signaling pathway.

Gene set enrichment analysis was performed to understand the meaningful pathway between gastritis and normal control. The top 20 significantly enriched pathways were displayed in Table 3. Of which, MAPK signaling pathway, cytokine-cytokine receptor interaction, chemokine signaling pathway, T cell receptor signaling pathway, and Toll-like receptor signaling pathway were displayed in Figure 8.

### 3.7. Real-Time Quantitative PCR Analysis

**Figure 9:**
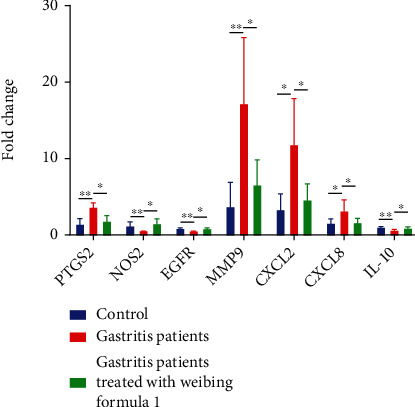
Validation candidate targets by qRT-PCR. The expression of candidate targets was detected by qRT-PCR assay. ∗ indicated *p* value <0.05, and ∗∗ indicated *p* value <0.01.

To determine the role of the candidate targets in the treatment of gastritis with Weibing Formula 1, the expressions of 7 genes (PTGS2, NOS2, EGFR, MMP9, CXCL2, CXCL8, and IL-10) were measured by quantitative real-time PCR in blood samples of normal control, gastritis patients, and gastritis patients treated with Weibing Formula 1. Among which, MMP9, CXCL2, CXCL8, and IL-10 were members of 15 common genes, and PTGS2, NOS2, and EGFR were members of 60 candidate targets, but do not belong to 15 common genes. Compared with the control group, the expressions of PTGS2, MMP9, CXCL2, and CXCL8 were significantly upregulated and NOS2, EGFR, and IL-10 were downregulated in gastritis patients, while the expressions of PTGS2, MMP9, CXCL2, and CXCL8 were significantly downregulated and NOS2, EGFR, and IL-10 were upregulated after the treatment of Weibing Formula 1 (Figure 9). Of which, MMP9, CXCL2, CXCL8, and IL-10 were part of the 15 common genes, and these four genes are significantly upregulated between gastritis patients and normal control. As shown in Figure 8, in addition to IL-10, MMP9, CXCL2, and CXCL8 expression levels were significantly increased in gastritis patients compared with the control group, which was consistent with the GSE60427 and GSE5081 results. These results suggest that Weibing Formula 1 may be involved in the treatment of gastritis by regulating these targets.

## 4. Discussion

Western drug is usually used for antibacterial therapy of gastritis and gastric-mucosa protection. However, western drug may cause bacterial resistance, with an increasing number of drugs failing to effectively control gastritis. Studies have shown that traditional Chinese medicine can achieve similar efficacy than western drug [25]. In China, traditional Chinese medicine, which includes Weibing Formula 1, has good therapeutic effects in gastritis patients of spleen-deficiency and Qi-stagnation syndrome. However, the pharmacological mechanism of Weibing Formula 1 in the treatment of gastritis is still incompletely understood. In the current study, we tried to combine network pharmacology and bioinformatics to discover the potential mechanism of Weibing Formula 1 in treating gastritis.

Network pharmacology is a powerful way for defining a variety of components and studying the mechanisms of Chinese herbal medicine [26]. In this study, we found that there were 135 active ingredients in Weibing Formula 1 according to the criteria of OB ≥ 30% and DL ≥ 0.18. After eliminating duplications, 251 potential targets corresponding to 135 bioactive components were obtained. Then, the DisGeNET, MalaCards, and CTD were used to uncover known gastritis-related targets, and 327 gastritis-related targets were retrieved. Among which, 60 common targets were shared between potential targets of Weibing Formula 1 and known gastritis-related targets. In addition, we structured the drug-active ingredients-genes–disease network and PPI network of 60 candidate targets. Furthermore, function enrichment analysis of 60 candidate targets was carried out using the David 6.8. Ultimately, the GEO database and real-time quantitative PCR were used to verify the related genes.

KEGG pathway enrichment analysis displayed that 60 common targets were markedly enriched in Toll-like receptor signaling pathway, NOD-like receptor signaling pathway, apoptosis, T cell receptor signaling pathway, MAPK signaling pathway, cytokine-cytokine receptor interaction pathway, and chemokine signaling pathway. Toll-like receptor signaling pathway, MAPK signaling pathway, and chemokine signaling pathway are the main pathways associated with Agastache rugosa treatment of gastritis [27]. The ethyl acetate extract of Alpinia officinarum Hance inhibits the development of Helicobacter pylori-associated gastritis by regulating MAPK signaling pathway [28]. Dang et al. have found that p53 upregulated modulator of apoptosis-mediated epithelial cell apoptosis promotes Helicobacter pylori infection-mediated gastritis [29]. Momordica charantia polysaccharides improve apoptosis in ethanol-induced gastritis in mucosa by inhibiting NF-*κ*B signaling pathway [30]. Quercetin protects against gastritis via regulating the balance of gastric cell proliferation and apoptosis [31]. During the progression of gastric disease, cytokine-cytokine receptor interaction pathway and chemokine signaling pathway are activated, triggering a chronic inflammatory response [2]. The above indicated that Toll-like receptor signaling pathway, MAPK signaling pathway, cytokine-cytokine receptor interaction pathway, chemokine signaling pathway, and apoptosis might play important roles in gastritis therapy. Furthermore, gene set enrichment analysis was also performed to understand the meaningful pathway between gastritis and normal control. Of which, MAPK signaling pathway, cytokine-cytokine receptor interaction, chemokine signaling pathway, T cell receptor signaling pathway, and Toll-like receptor signaling pathway were significantly enriched pathway. Therefore, we speculated that Weibing Formula 1 might participate in the treatment of gastritis by regulating these pathways.

Epidermal growth factor (EGF) is one of the fatal factors in gastric tissue repair and cell regeneration. It binds to its receptor epidermal growth factor receptor (EGFR) located in the basement membrane or bilateral membrane of gastric epithelium. EGFR is highly expressed in damaged gastric barrier and perfusion epithelial cells, but rarely in normal epithelial cells. Studies have shown that the expression of EGFR in the surface mucosa of the stomach is higher than that in the deep and muscle layers of the stomach, which indicates its role in promoting the growth and recovery of damaged gastric epithelial cells [32]. It has also been reported that high expression of EGFR can induce malignant growth of gastric epithelial cells and lead to a certain degree of canceration [33]. Chronic atrophic gastritis rats may participate in gastric mucosal barrier injury, cell proliferation, and apoptosis by regulating the expression of EGFR and Wei-Wei-Kang-Granule against gastritis by decreasing the express of EGFR in gastric mucosa [34]. Our real-time quantitative results showed that the expression of EGFR in gastritis patients was significantly upregulated, while the expressions of EGFR were significantly downregulated after the treatment of Weibing Formula 1. In addition, EGFR was significantly enriched in MAPK signaling pathway and cytokine-cytokine receptor interaction pathway. Therefore, we inferred that Weibing Formula 1 might be involved in the progression of gastritis by regulating EGFR activation MAPK signaling pathway and cytokine-cytokine receptor interaction pathway.

GSE60427 was used to identify differentially expressed genes, and we obtained 895 differentially expressed genes between gastritis and normal control. A total of 15 common genes were shared between differentially expressed genes and 60 candidate targets. Among which, interleukin- (IL-) 6 (IL-6) and CC-chemokine ligand 2 (CCL2), as members of 15 common genes, have been reported to be associated with the progression of gastritis. IL-6 is a multipotent four-helix cytokine that performs a variety of functions in the body [35]. IL-6 can be secreted by T cells and macrophages, causing an immune response during infection or after injury and ultimately leading to inflammation of the gastric mucosa or other tissues [36]. Recently, IL-6 is reported to play an important role in the treatment of chronic gastritis in Zuojinwan [2]. Moreover, IL-6 has been reported to be involved in the treatment of gastritis by Atractylodes Macrocephala Koidz [1]. CCL2, also named as MCP-1, is a micromolecular effective chemoattractant for leukocytes. A study has shown that overexpression of CCL2 may be closely related to gastric diseases, which is considered to be predictive molecules of gastric cancer [37]. The verification results of the GSE5081 dataset displayed that IL-6 and CCL2 were significantly upregulated in gastritis compared to normal, which was consistent with the results in the GSE60427 dataset. Thence, we postulated that IL-6 and CCL2 might serve as the key points for the treatment of gastritis in the Weibing Formula 1.

In conclusion, the potential mechanism of Weibing Formula 1 in treating gastritis has the multicomponent, multitarget, and multiway characteristics, and PTGS2, NOS2, EGFR, MMP9, CXCL2, CXCL8, and IL-10 may be the important direct targets of Weibing Formula 1 in gastritis treatment. Such data provide the basis for multi-ingredient synergies in future research. Our study revealed the mechanism of Weibing Formula 1 in gastritis from an overall and systematic perspective, providing a theoretical basis for further knowing and application of this formula in the future. However, whether Weibing Formula 1 is suitable for long-term maintenance treatment of gastritis still needs to be confirmed in future clinical trials. Thence, it is necessary to conduct further basic experimental verification of the potential active ingredients in order to clarify the theoretical prediction.

## Figures and Tables

**Table 1:** The top 10 active compounds in Weibing Formula 1.

Molecule ID	Molecule name	OB (%)	DL	Molecular structure	Herb	Number of targets
MOL000098	Quercetin	46.43	0.28	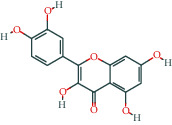	Plantaginis Herba/Crataegus pinnatifida Bunge/Coptidis Rhizoma/Licorice	152
MOL000422	Kaempferol	41.88	0.24	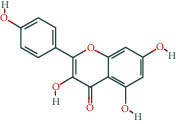	Crataegus pinnatifida Bunge/Paeoniae Radix Alba/Licorice	62
MOL003896	7-Methoxy-2-methyl isoflavone	42.56	0.2	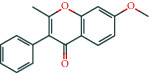	Licorice	43
MOL000392	Formononetin	69.67	0.21	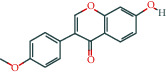	Licorice	39
MOL000358	Beta-sitosterol	36.91	0.75	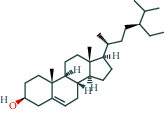	Paeoniae Radix Alba/Aurantii Fructus	37
MOL004328	Naringenin	59.29	0.21	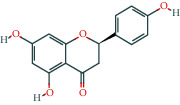	Aurantii Fructus/Licorice	37
MOL000354	Isorhamnetin	49.6	0.31	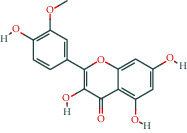	Crataegus pinnatifida Bunge/Licorice	36
MOL005828	Nobiletin	61.67	0.52	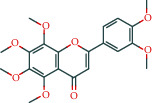	Aurantii Fructus	35
MOL002565	Medicarpin	49.22	0.34	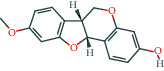	Licorice	34
MOL000449	Stigmasterol	43.83	0.76	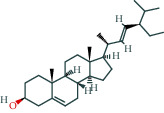	Crataegus pinnatifida Bunge/Aucklandiae Radix/Herba Taraxaci	32

**Table 2:** The top 20 differentially expressed genes between gastritis and normal control.

Symbol	logFC	AveExpr	*p* value	FDR value	Up/down
FCRL2	3.676252	6.396535	1.29*E*-14	2.81*E*-10	Up
CD180	3.681265	4.779778	5.03*E*-14	3.87*E*-10	Up
CCL20	4.643928	9.7374	5.34*E*-14	3.87*E*-10	Up
ADAMDEC1	3.547777	6.194662	6.49*E*-13	2.82*E*-09	Up
CD19	5.24656	8.950685	6.50*E*-13	2.82*E*-09	Up
CXCL1	3.459952	9.175125	1.03*E*-12	3.74*E*-09	Up
PLA2G7	3.659776	7.439826	1.95*E*-12	6.04*E*-09	Up
CXCL2	3.227429	9.547699	2.35*E*-12	6.37*E*-09	Up
LCN2	3.91543	13.39285	3.50*E*-12	8.45*E*-09	Up
FCRL5	2.80746	6.40599	5.25*E*-12	1.07*E*-08	Up
NMU	-1.54903	11.76094	2.10*E*-08	2.28*E*-06	Down
PRB2	-1.69213	2.736936	1.03*E*-07	7.44*E*-06	Down
FABP5	-1.03799	12.32549	1.82E-07	1.15E-05	Down
C6	-3.59553	7.529899	2.51*E*-07	1.49*E*-05	Down
EDA	-1.10261	6.242344	3.97*E*-07	2.20*E*-05	Down
FAM174B	-1.0484	11.10483	5.76*E*-07	2.90*E*-05	Down
DBP	-1.65056	8.455515	9.36*E*-07	4.38*E*-05	Down
MPV17L	-1.17795	6.826452	1.06*E*-06	4.81*E*-05	Down
IGFBP4	-1.07855	8.68517	1.27*E*-06	5.67*E*-05	Down
RASL11B	-1.34426	7.673157	2.53*E*-06	9.82*E*-05	Down

**Table 3:** The top 20 significantly enriched pathway between gastritis and normal control.

Name	Size	ES	NES	NOM *p* val	FDR *q* val	FWER *p* val
Pathways in cancer	322	-0.444	-1.662	*p* ≤ 0.001	0.051	0.402
MAPK signaling pathway	264	-0.403	-1.47	0.004	0.104	0.841
Cytokine-cytokine receptor interaction	252	-0.706	-1.703	*p* ≤ 0.001	0.043	0.313
Regulation of actin cytoskeleton	211	-0.362	-1.396	0.03	0.142	0.913
Focal adhesion	197	-0.463	-1.491	0.045	0.09	0.804
Chemokine signaling pathway	183	-0.747	-1.793	*p* ≤ 0.001	0.039	0.146
Endocytosis	176	-0.407	-1.86	0.004	0.028	0.079
Jak Stat signaling pathway	147	-0.572	-1.731	*p* ≤ 0.001	0.044	0.247
Alzheimer's disease	143	-0.384	-1.463	0.032	0.107	0.848
Insulin signaling pathway	137	-0.356	-1.45	0.046	0.115	0.864
Cell adhesion molecules (CAMs)	130	-0.701	-1.652	*p* ≤ 0.001	0.053	0.425
Natural killer cell-mediated cytotoxicity	124	-0.634	-1.707	*p* ≤ 0.001	0.044	0.304
Neurotrophin signaling pathway	124	-0.407	-1.624	0.011	0.052	0.502
Leukocyte transendothelial migration	115	-0.645	-1.686	*p* ≤ 0.001	0.044	0.348
Vascular smooth muscle contraction	112	-0.468	-1.431	0.016	0.127	0.884
T cell receptor signaling pathway	106	-0.641	-1.758	*p* ≤ 0.001	0.043	0.2
Melanogenesis	101	-0.414	-1.409	0.032	0.135	0.899
GnRH signaling pathway	100	-0.439	-1.528	0.01	0.071	0.734
Parkinson's disease	98	-0.41	-1.584	0.025	0.065	0.615
Toll-like receptor signaling pathway	96	-0.681	-1.77	*p* ≤ 0.001	0.042	0.175

## Data Availability

The data used to support the findings of this study are included within the article.
